# Isolation and in vitro culture of endothelial progenitor cell-like populations from leukoreduction filters

**DOI:** 10.1186/s12896-026-01209-y

**Published:** 2026-08-10

**Authors:** Zahra Abbasi-Malati, Seyed Ghader Azizi, Sahar Balagholi, Ali Akbar Pourfathollah

**Affiliations:** 1 Blood Transfusion Research Center, High Institute for Research and Education in Transfusion Medicine, Tehran, Iran; 2 Department of Tissue Engineering and Applied Cell Sciences, School of Paramedicine, Guilan University of Medical Sciences, Rasht, Iran; 3 Clinical Immunology Research Center, Zahedan University of Medical Sciences, Zahedan, Iran; 4 Department of Immunology, Faculty of Medical Sciences, Tarbiat Modares University, Tehran, 14115-111 Iran

**Keywords:** Leukoreduction filters, Endothelial progenitor cell-like populations, Cell isolation, In vitro culture, Blood-derived cells

## Abstract

**Background:**

Leukoreduction filters (LRFs) are routinely used in blood transfusion services and retain substantial numbers of nucleated cells during blood processing. These discarded filters have therefore been explored as a potential source of peripheral blood-derived cells for endothelial progenitor cell (EPC)-like related in vitro studies.

**Methods:**

Leukoreduction filters were collected from the Iranian Blood Transfusion Organization (IBTO). Entrapped cells were recovered using a washing-based procedure without the use of magnetic-activated cell sorting (MACS) or endothelial differentiation media. A coating solution containing plasma-derived components, including cryoprecipitate and platelet lysate, was applied to improve early cell adhesion during in vitro culture.

**Results:**

Cultured cells were characterized by flow cytometry using CD133 as a surface-associated progenitor marker. Approximately 70% of the cells at passage 3–4 was positive for CD133.

**Conclusion:**

Leukoreduction filters may serve as an accessible and cost-effective source of peripheral blood-derived cell populations for EPC-like related in vitro studies. Further studies are required to confirm their endothelial identity and functional properties.

## Introduction

In recent years, endothelial progenitor cells (EPCs), a heterogeneous population of progenitor cells associated with vascular repair and angiogenesis, have attracted considerable attention in regenerative medicine, particularly cardiovascular research. EPCs have been described as heterogeneous mononuclear cell populations originating from hematopoietic and vascular progenitor compartments [1]. These cells, also referred to as vascular endothelial progenitor-like populations in some studies, represent a small fraction of bone marrow-derived cells and have been reported to participate in angiogenic processes [2–4]. Several surface markers have been proposed for the identification of EPC populations. Commonly reported markers include CD34 and vascular endothelial growth factor receptor-2 (VEGFR-2); however, EPC characterization remains challenging due to the lack of a single specific marker and the phenotypic heterogeneity of reported EPC populations. Notably, CD34⁺ cells represent a low proportion of mononuclear cells in the peripheral blood of healthy individuals [5]. 

The potential application of EPC-related populations in cell-based therapies has increased interest in their biological properties. EPCs have been investigated for their potential contribution to tissue repair, neovascularization, and paracrine signaling in experimental models [3]. 

However, the low frequency of EPC-related populations in peripheral blood presents a significant challenge, making their isolation and expansion a complex and time-consuming process. The limited presence of these populations in the peripheral has been attributed to factors including restricted progenitor cell mobilization, depletion of progenitor pools, and increased apoptosis [6]. Moreover, certain risk factors such as diabetes, aging, atherosclerosis, and hypertension have been reported to reduce circulating EPC-related populations by hampering their mobilization and increasing their apoptosis [7]. Despite these challenges, EPC-related populations remain of interest due to their reported endothelial- associated properties, including angiogenic factor secretion and endothelial-like functional responses.

Several limitations associated with current EPC sources include ethical considerations and practical constraints related to cell availability and expansion. In this regard, exploring alternative cell sources may help overcome limitations associated with traditional EPC sources, including restricted availability and expansion capacity. Recently, leukoreduction filters have been explored as a potential source of peripheral blood–derived cells for EPC-related research [8]. It should be noted that culture, expansion, and purification methods require further optimization. Leukoreduction of blood products before storage is a routine procedure used to reduce transfusion-related complications, including reactions associated with antibodies against HLA, viral infections, febrile non-hemolytic transfusion reactions (FNHTRs), and platelet refractoriness. This procedure is performed using leukoreduction filters (LRFs), which retain leukocytes based on physical filtration properties [9]. 

Cells recovered from leukoreduction filters have attracted attention as a potential resource for EPC-related in vitro studies. LRFs can retain various peripheral blood-derived cells, including mononuclear cells and progenitor-like populations, which are usually discarded. Cells retained within leukoreduction filters may represent a useful source of peripheral blood–derived cells for in vitro investigations. The purpose of this study was to isolate and expand endothelial progenitor cell-like populations from leukoreduction filters using a practical and cost-effective approach.

## Materials and methods

### Filter supply and ethical approval

The use of discarded leukoreduction filters for research purposes was approved by the Ethics Committee of the Iranian Blood Transfusion Organization (IBTO)(Ethics code: IR.TMI.REC.1397.016). The blood collection process was performed according to the standard protocols of IBTO, and no additional intervention was performed by the investigators.

### Filter elution and mononuclear cell isolation from leukoreduction filters

1.  **Preparation of elution buffer**

Elution buffer composed of phosphate-buffered saline (PBS) containing 5 mM disodium EDTA (EDTA-Na₂) and 2.5% (w/v) sucrose was prepared based on the formulation described by Meyer et al. [10] and subsequently stored at 4 °C.

2. **Leukoreduction filter washing**

**Fig. 1:**
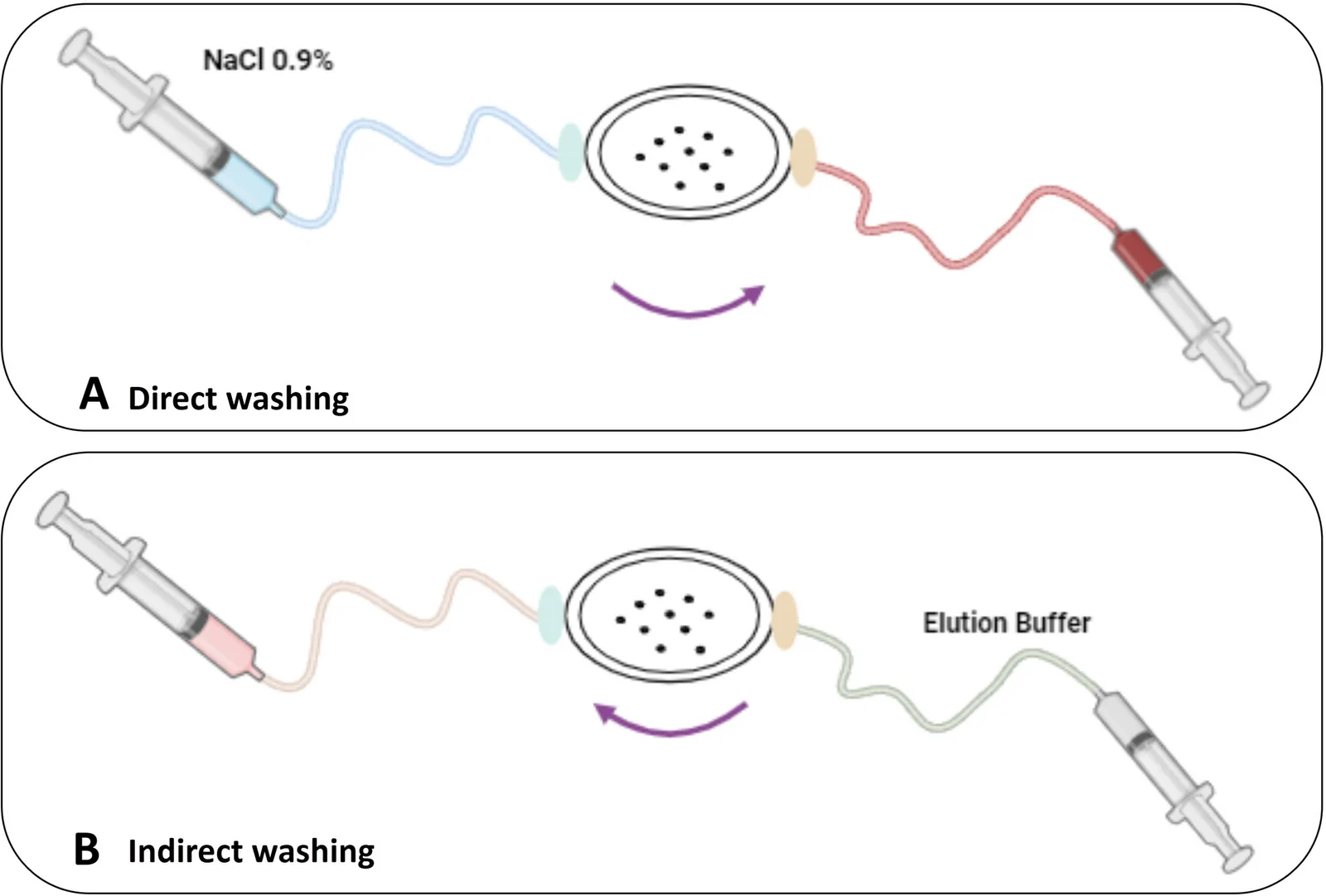
Schematic representation of the leukoreduction filter washing procedure. (**A**) Direct washing performed in the original direction of blood filtration to reduce residual red blood cells. (**B**) Indirect washing performed in the reverse direction of filtration to elute retained nucleated cells

A previously described washing procedure was used to elute nucleated cells from leukoreduction filters [8]. Briefly, a double-syringe and multi-step washing system was used for cell extraction. The first step, referred to as direct washing (reduction of residual red blood cells), involves flushing the filter in the direction of blood filtration with a 0.9% NaCl solution (Fig. 1A). The second step, indirect washing, involved flushing the filters with elution buffer against the direction of filtration. Leukocytes were released by mechanical forces and suction (Fig. 1B).

### Pre-processing and density gradient cell separation

The solution obtained from indirect washing was centrifuged (Eppendorf, Germany) at 800 × g for 15 min, and this step was repeated three times to reduce platelet-derived particles and debris generated during the washing process. After each centrifugation step, the supernatant was carefully decanted, and 50 mL of elution buffer was added for resuspension. The resulting mixture was then centrifuged at 2000 × g for 15 min.

**Fig. 2:**
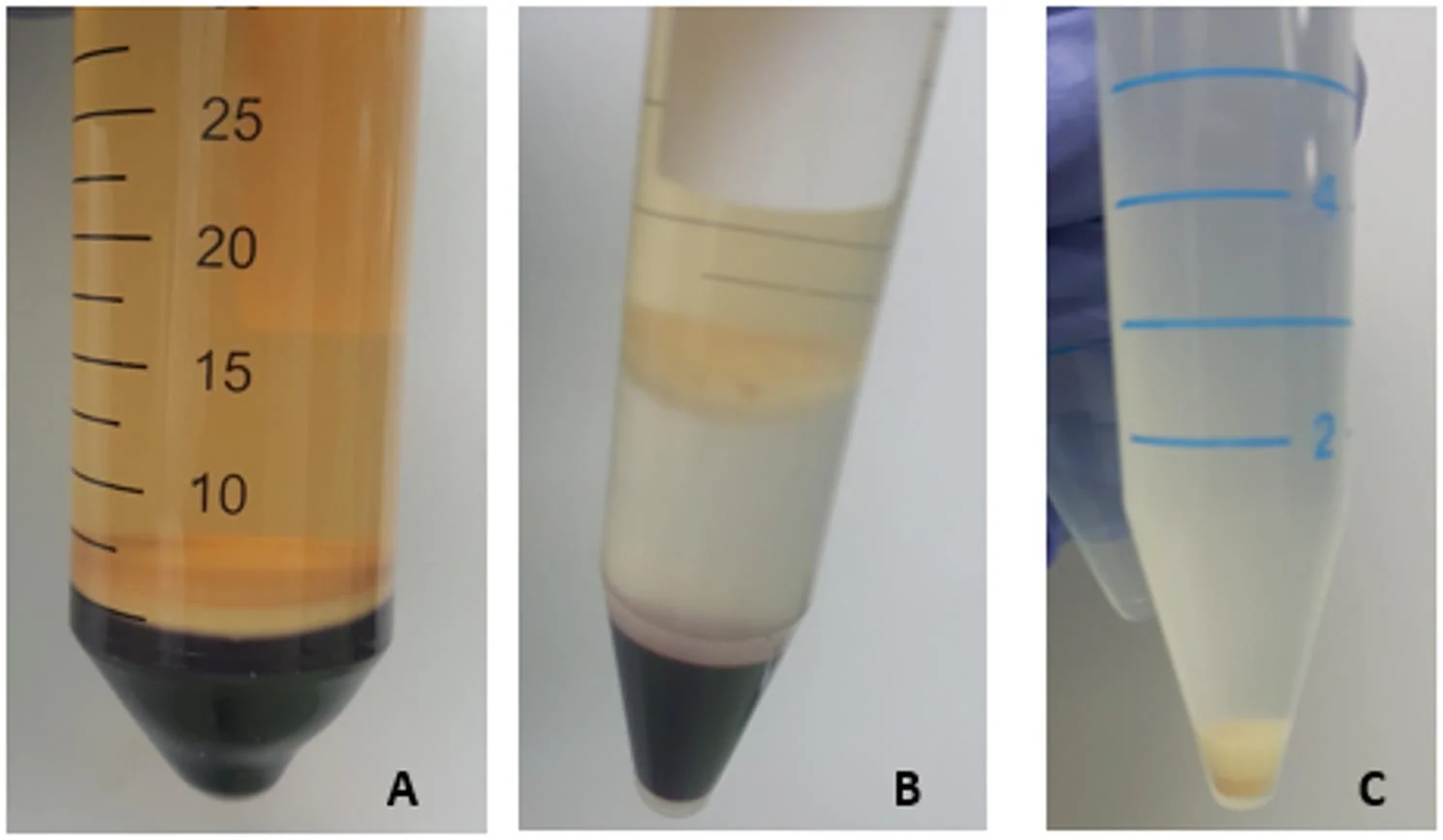
Pre-processing and density gradient separation of mononuclear cells. (**A**) Collection of the intermediate white layer containing mononuclear cells after centrifugation. (**B**) Isolation of mononuclear cells at the interface following density gradient centrifugation. (**C**) Harvested mononuclear cells after washing with PBS

The tube contents were fractionated into three distinct layers following the centrifugation process. The intermediate white layer containing mononuclear cell–enriched fraction was carefully aspirated using Pasteur pipette and transferred to a separate tube, as illustrated in Fig. 2A [8]. 

MNCs were isolated using a density gradient medium (Inno-train, Germany) at a 2:1 sample-to-medium volume ratio. The procedure was performed gently to avoid mixing of the layers. The tube was centrifuged at 800 x g for 25 min with the brake turned off. After the centrifugation, the tube’s contents were fractionated into three layers (Fig. 2B). The MNCs were carefully harvested from the interface layer using a Pasteur pipette. The harvested cells were washed twice with PBS (Fig. 2C).

### Culture of endothelial progenitor cell-like populations

#### Preparation of cryoprecipitate

Fresh frozen plasma (FFP) was procured to facilitate the preparation of cryoprecipitate. The cryoprecipitation process adhered to the standard IBTO protocol. Each cryoprecipitate unit was prepared from a minimum of 200 mL of FFP. Upon thawing the FFP at 4 °C, a white precipitate, referred to as cryoprecipitate (Cryo), was formed. Subsequently, the thawed plasma underwent centrifugation at 4 °C, wherein the cryo-poor plasma (CPP) was extracted and transferred to the satellite bag, while a residue of approximately 10 to 15 mL remained on the Cryo deposit.

Cryoprecipitate contains plasma-derived proteins, including fibrinogen, factor VIII, von Willebrand factor, factor XIII, and other plasma-associated extracellular matrix proteins such as fibronectin. In this study, cryoprecipitate was used as a plasma-derived coating component to support cell attachment during culture.

1. **Preparation of platelet lysate with freeze-thaw (FT) process**

The Innovation Center of IBTO processed platelet bags after obtaining consent from the donors. Platelet lysate was generated using repeated freeze–thaw (FT) cycles. Specifically, the platelet suspension underwent at least 5 h of freezing at -80 °C, followed by defrosting. This process was iterated three times, followed by centrifugation of the lysate at 2800 × g for 15 min to collect the supernatant containing platelet lysate.

2. **Preparation of** **coating solution**

A 5% cryoprecipitate and 95% platelet lysate coating solution was prepared by combining 950 µL platelet lysate with 50 µL cryoprecipitate. The coating formulation was selected based on the presence of plasma-derived adhesion-supporting components.

3. **EPCs** **culture**

To initiate culture of EPC-like populations, culture flasks were incubated with the coating solution for one hour at 37 °C in a humidified atmosphere containing 5% CO₂. Following two washes with 1× phosphate-buffered saline (PBS), the isolated mononuclear cell (MNC) fraction was resuspended in 1 mL of culture medium and transferred into the coated flask.

After 24 h of incubation, the supernatant containing non-adherent cells was removed, and fresh culture medium supplemented with 5% platelet lysate was added to the adherent cell population. During medium replacement, the existing medium was removed, and the cells were gently washed once with a small volume of 1× PBS. Fresh medium containing 5% platelet lysate was then added. Cell passage was performed when the adherent cells reached approximately 80% confluence. Cells were detached using trypsin-EDTA (0.25%) according to standard cell culture procedures.

### Characterization of endothelial progenitor cell-like populations

After isolation and culture of endothelial progenitor cell-like populations recovered from leukoreduction filters, cell morphology was examined using an inverted microscope 10× and 20× magnifications (Nikon, Japan at).

Cell samples collected at passage 3 were analyzed by flow cytometry for CD133 expression. To be specific, the cell suspension was partitioned into five tubes, each containing 5 × 10⁵ cells. Subsequently, 5 µl of CD133-PE conjugated monoclonal antibody (BioLegend, USA) was added to each tube and incubated at 4 °C in darkness for 30 min. Under such conditions, the cells were washed with 500 µl of PBS and fixed with 50 µl of 1% paraformaldehyde. An unstained control was used for gating. The resulting fixed cells were then evaluated and compared to the control group utilizing the PARTEC flow cytometry device and FlowJo v10 software.

### Investigating the growth kinetics of endothelial progenitor cell-like populations

A growth curve was generated to evaluate the in vitro expansion pattern of endothelial progenitor cell-like populations. The cell number was determined at passage 4 during the logarithmic growth phase using an automated cell counter (Sysmex KX21) in three independent wells. Subsequently, 2 × 10⁴ cells were seeded into each well of a 6-well plate in triplicate. Cells were detached using trypsin-EDTA every 24 h for seven consecutive days and counted at each time point.

Doubling time (DT) was calculated as: $\:DT=\frac{T\times\:ln\left(2\right)}{In\:\left(\frac{Xt}{X0}\right)}$

where DT represents the doubling time, T represents the incubation time, X₀ represents the initial cell number, and Xt represents the final cell number after incubation. The growth curve was generated using Microsoft Excel, and the population doubling time was calculated during the logarithmic phase.

### Assessment of endothelial-associated network formation on Matrigel

To evaluate the endothelial-associated organizational behavior of the cultured cell population, a Matrigel-based assay was performed. Briefly, cells at passage 3 were seeded onto Matrigel-coated culture wells and incubated at 37 °C in a humidified atmosphere containing 5% CO₂. After 24 h of incubation, cell morphology and formation of network-like structures were evaluated using an inverted phase-contrast microscope. Representative images were captured from three independent wells were analyzed. The extent of network formation was subsequently analyzed using ImageJ software.

## Results

### Isolation and expansion of endothelial progenitor cell-like populations from leukoreduction filters

Cells displaying endothelial progenitor cell-like characteristics were recovered from discarded leukoreduction filters using a washing-based isolation approach and expanded under platelet lysate–supplemented culture conditions. This approach allowed the maintenance and expansion of adherent cell populations without the use of endothelial differentiation media.

### Morphological characterization of endothelial progenitor cell-like populations

Following isolation and culture of leukoreduction filter-derived endothelial progenitor cell-like populations, cellular morphology was evaluated using an inverted microscope.

**Fig. 3:**
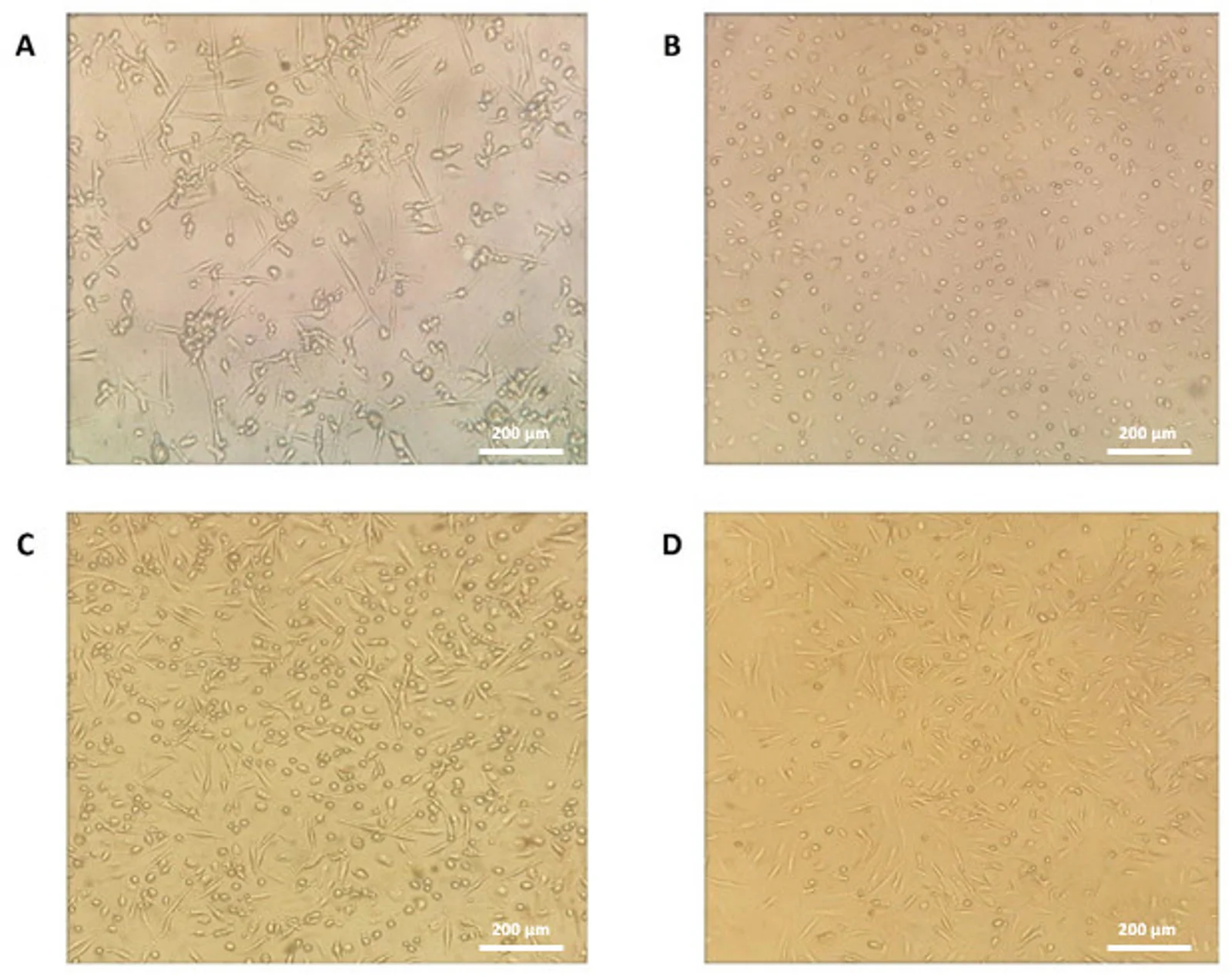
Morphological evaluation of leukoreduction filter-derived endothelial progenitor cell-like populations during early culture. (**A**) Representative phase-contrast image of cells cultured on cryoprecipitate-coated flasks after 15 days using pooled cells recovered from five leukoreduction filters. (**B**) Representative image of cells cultured on cryoprecipitate and platelet lysate-coated flasks after 3 days using cells recovered from a single leukoreduction filter. (**C**) Representative image of adherent cells under the same culture conditions after 7 days. (**D**) Early attachment of adherent cells observed after 24 h of culture. Images demonstrate the emergence of adherent cell populations under the applied culture conditions. Scale bars and magnification information are provided in the figure panels

**Fig. 4:**
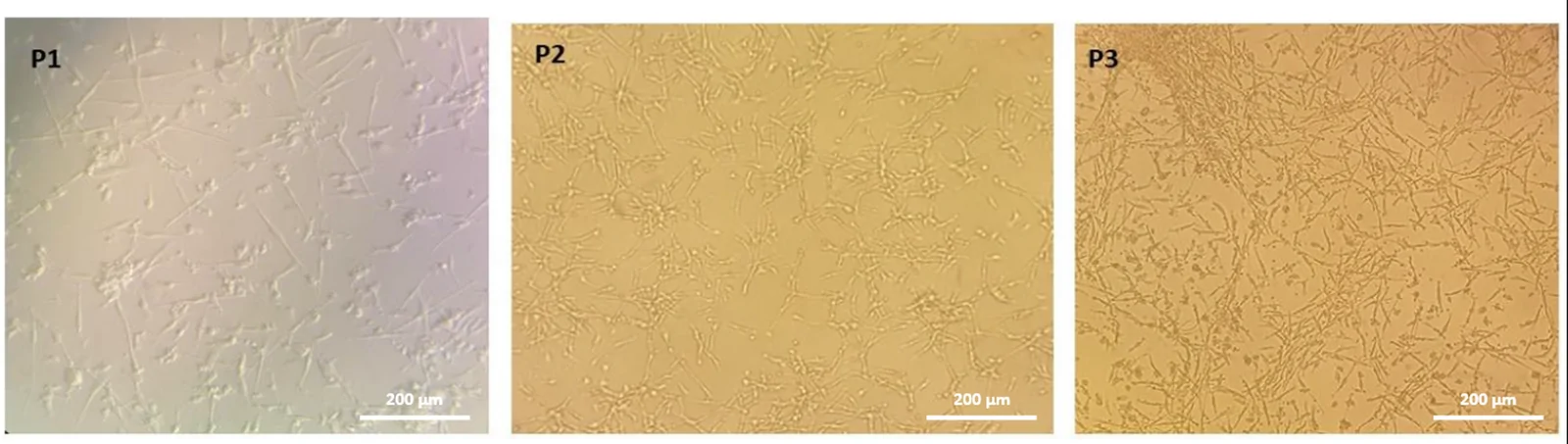
Morphological changes of leukoreduction filter-derived endothelial progenitor cell-like populations during consecutive passages. Representative phase-contrast images showing adherent cells during in vitro expansion. The spindle-shaped morphology observed in culture represents an endothelial-associated progenitor-like phenotype but is not considered specific for EPC identity

Initially, most cells exhibited a rounded morphology and remained non-adherent in the culture medium. After the first day of culture, adherent cells were observed on the coated culture surface, suggesting attachment and survival of a subpopulation under the applied culture conditions. Further microscopic examination during subsequent passages showed that the expanded adherent cells predominantly exhibited a spindle-shaped morphology, which is commonly observed in several adherent progenitor-like and endothelial-associated cell cultures. Representative images are presented in Figs. 3 and 4.

### Assessment of CD133 expression in endothelial progenitor cell-like populations

The cultured endothelial progenitor cell-like populations were assessed for CD133-PE expression using flow cytometry.

**Fig. 5:**
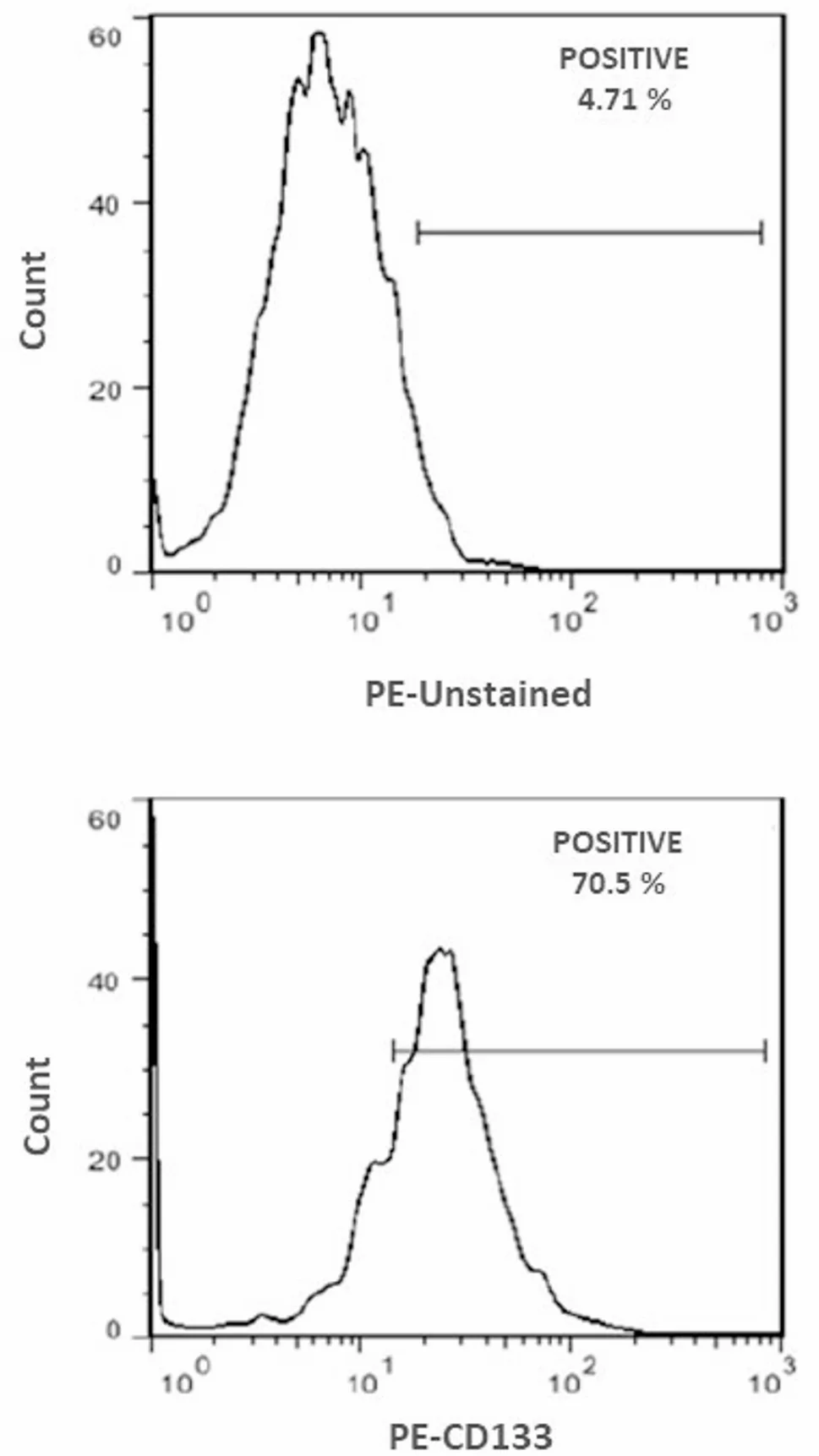
Flow cytometric assessment of CD133 expression in leukoreduction filter-derived endothelial progenitor cell-like populations at passage 3. Representative histogram showing CD133-positive cells compared with unstained control. CD133 expression was evaluated as a progenitor-associated marker and was interpreted together with other phenotypic and functional observations

Flow cytometry analysis demonstrated that approximately 70% of cultured cells at passage 3 showed CD133 positivity (Fig. 5). Unstained cells were used as a negative control for background fluorescence assessment.

### Growth kinetics of endothelial progenitor cell-like populations

**Fig. 6:**
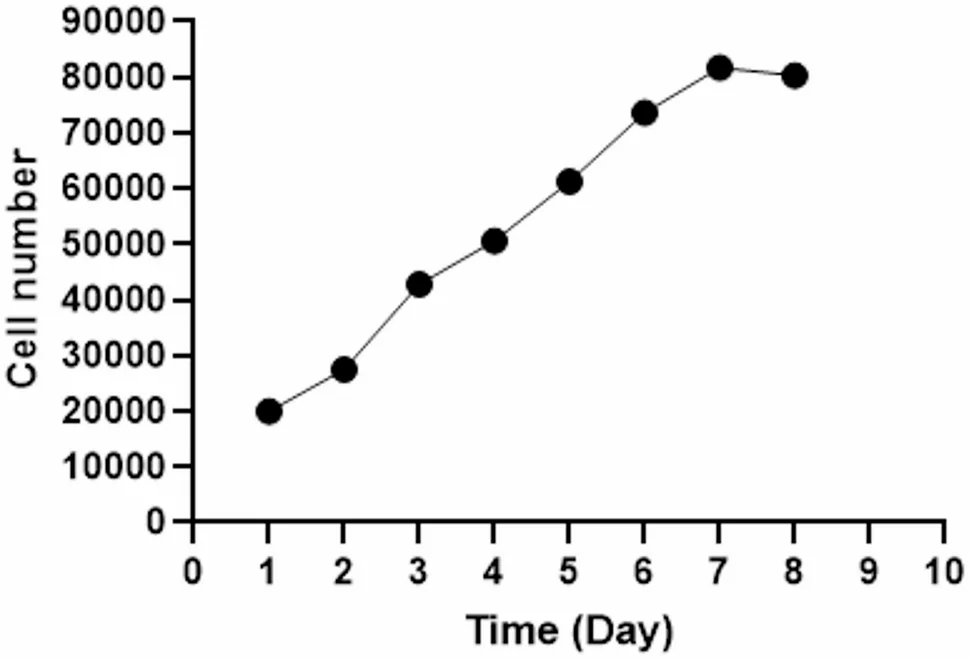
Growth kinetics of cultured cells derived from leukoreduction filters. Cells were counted daily for seven days following seeding, and the growth curve was generated based on cell numbers over time. Doubling time was calculated during the logarithmic growth phase

Growth curve analysis demonstrated the in vitro expansion pattern of endothelial progenitor cell-like populations during culture. The cells showed an initial lag phase followed by an exponential growth phase, after which the proliferation rate gradually decreased and reached a plateau phase (Fig. 6). The population doubling time was calculated during the logarithmic growth phase. Quantitative analysis was performed using three independent wells, and data are presented as mean ± SD.

### Assessment of endothelial-associated network formation on matrigel

**Fig. 7:**
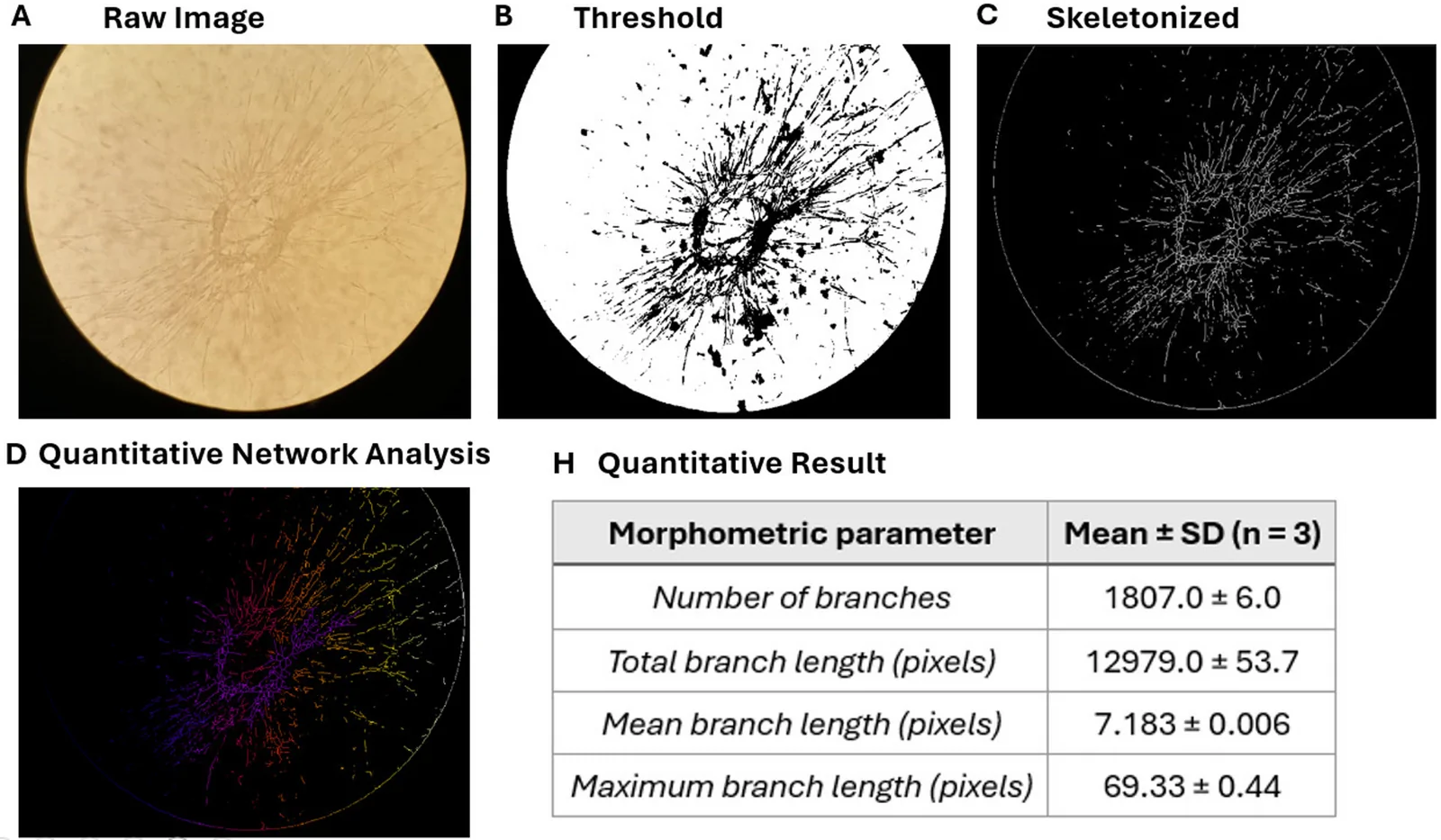
Assessment of endothelial-associated network formation on Matrigel. Representative images showing evaluation and quantitative analysis of network-like structures formed under Matrigel culture conditions. (**A**) Representative phase-contrast image showing endothelial-associated network-like structures formed by cultured leukoreduction filter-derived cell populations. (**B**) Binary thresholded image generated for image segmentation. (**C**) Skeletonized representation of detected network structures used for morphometric analysis. (**D**) Quantitative analysis of skeletonized structures based on network architecture parameters, including number of branches, total branch length, average branch length, and maximum branch length. Data are presented as mean ± SD from analyzed fields

To further evaluate endothelial-associated behavior, cultured endothelial progenitor cell-like populations were assessed using a Matrigel-based network formation assay. Following incubation on Matrigel, the cells demonstrated the formation of interconnected network-like structures under culture conditions (Fig. 7). Quantitative analysis of network formation parameters was performed using Fiji/ImageJ skeleton analysis, including the number of branches, total branch length, and average branch length. The analyzed fields showed a mean number of 1807.0 ± 6.0 branches, with a total branch length of 12979.0 ± 53.7 pixels. These findings indicate endothelial-associated network-forming behavior of the cultured cell population under Matrigel conditions.

## Discussion

As described previously, the isolation and characterization of EPC-like populations present numerous challenges. A primary obstacle is the absence of a standardized definition for EPCs, which complicates the comparison of results among different studies. The heterogeneity of reported EPC populations and the lack of highly specific markers further complicate their isolation and characterization [11]. 

In addition, previous studies have suggested that some EPC-derived populations may exhibit limited lifespan and reduced proliferative capacity during in vitro expansion compared with other stem/progenitor cell populations.

Such observations have raised concerns regarding the ability to obtain sufficient cell numbers of cells for experimental and therapeutic applications [12]. Therefore, limitations related to cell availability, expansion capacity, and culture stability remain important considerations for the potential application of EPC-based approaches [13].

Despite these challenges, substantial progress has been made in understanding the biological properties of EPC-like populations. Preclinical investigations have suggested angiogenic activity and paracrine effects associated with EPC-related populations in experimental models of ischemic diseases [14]. A noteworthy obstacle is the low frequency of EPC-related populations in peripheral blood, rendering their isolation and identification time-consuming and technically demanding [15]. Consequently, leukoreduction filters have been explored as an alternative source of peripheral blood-derived cells for the isolation and expansion of EPC-like populations, as suggested by previous reports.

Over the past decade, leukoreduction filters have been investigated as an accessible source of peripheral blood-derived cells for research applications.

Previous studies have reported that leukoreduction filters can retain higher numbers of CD34⁺ cells compared with peripheral blood samples [5]. The use of leukoreduction filters for research purposes requires ethical consideration and appropriate institutional approval. However, relatively few studies have specifically investigated the isolation and expansion of EPC-related populations from leukoreduction filters.

In a previous study, filter-derived peripheral blood mononuclear cells (PBMNCs) were cultured under specific conditions, and an increase in EPC-related cell expansion compared with fresh blood-derived samples was reported [16]. Furthermore, previous studies have reported higher numbers of EPC-related or progenitor-like cells recovered from leukoreduction filters compared with fresh peripheral blood samples over time [16]. 

These findings highlight the potential of leukoreduction filters as an alternative source for recovering rare peripheral blood-derived progenitor populations. A previous study compared the yield of EPC-related cell populations recovered from a leukoreduction filter (5.4 million) with that obtained from a fresh blood sample (0.4 million) [16].

In the present study, a two-syringe, multi-step washing procedure was employed to improve the recovery of retained cells from leukoreduction filters through: (1) reduction of residual red blood cells (RBCs) (2), release of trapped leukocytes, and (3) reduction of debris and microparticles generated during processing. Subsequently, a simple and cost-conscious culture approach was evaluated without the use of specific cell isolation techniques such as magnetic-activated cell sorting (MACS). In addition, endothelial differentiation media were not used in order to evaluate whether the recovered population could be expanded under simplified culture conditions. Given the limited initial attachment observed after isolation, a fibronectin-containing cryoprecipitate-based coating solution was used to support early cell adhesion during the initial culture period.

As observed in the initial culture period, cells recovered directly from leukoreduction filters exhibited a relatively slow expansion rate. Therefore, optimization of culture conditions was required to obtain sufficient numbers of adherent cell populations for subsequent characterization. During cell isolation and processing, the presence of platelet-derived components and plasma proteins required careful optimization of the processing steps to minimize aggregation and improve recovery of the target cell population.

In preliminary optimization experiments, cells from multiple filters were considered to increase the initial cell yield; however, the final experimental workflow was performed using individually processed filters. The use of platelet lysate as a culture supplement supported cell attachment and expansion under the applied culture conditions.

Overall, leukoreduction filters represent a potential and accessible source of peripheral blood-derived cells for EPC-related in vitro investigations. However, further studies using comprehensive immunophenotypic characterization and functional assays are required to clarify the endothelial identity and biological properties of these cell populations. The possibility of contamination or contribution from other CD133-expressing hematopoietic populations cannot be excluded based on the current characterization strategy.

## Data Availability

The datasets used and/or analyzed during the current study are available from the corresponding author on reasonable request.

## Abbreviations

EPC: Endothelial progenitor cell
LRF: Leukoreduction filter
IBTO: Iranian Blood Transfusion Organization
MACS: Magnetic-activated cell sorting
PBMC: Peripheral blood mononuclear cell
MNC: Mononuclear cell
PBS: Phosphate-buffered saline
EDTA: Ethylenediaminetetraacetic acid
NaCl: Sodium chloride
FFP: Fresh frozen plasma
CPP: Cryo-poor plasma
FT: Freeze–thaw
VWF: Von Willebrand factor
RBC: Red blood cell
HLA: Human leukocyte antigen
FNHTR: Febrile non-hemolytic transfusion reaction
